# Risk factors and outcomes of IgA nephropathy recurrence after kidney transplantation: a systematic review and meta-analysis

**DOI:** 10.3389/fimmu.2023.1277017

**Published:** 2023-11-28

**Authors:** Yue Li, Yangming Tang, Tao Lin, Turun Song

**Affiliations:** ^1^ Department of Urology, West China Hospital, Sichuan University, Chengdu, China; ^2^ Transplant Center, West China Hospital, Sichuan University, Chengdu, China

**Keywords:** IgA nephropathy, risk factors, kidney transplantation, recurrence, graft survival, systematic review

## Abstract

**Background:**

IgA nephropathy may recur in patients receiving kidney transplantation due to IgA nephropathy induced renal failure. The risk factors for recurrence are still at issue. The aim of this study was to conduct a systematic review and meta-analysis to assess risk factors and outcomes for IgA nephropathy recurrence.

**Methods:**

We used PubMed, EMBASE, Cochrane Library, Web of Science, Scopus, CNKI, WanFang, VIP and CBM to search for relevant studies published in English and Chinese. Cohort or case-control studies reporting risk factors or outcomes for IgA nephropathy recurrence were included.

**Results:**

Fifty-eight studies were included. Compare to no recurrence group, those with IgAN recurrence had younger age (mean difference [MD]=-4.27 years; risk ratio [RR]=0.96), younger donor age (MD=-2.19 years), shorter time from IgA nephropathy diagnosis to end stage renal disease (MD=-1.84 years; RR=0.94), shorter time on dialysis (MD=-3.14 months), lower human leukocyte-antigen (HLA) mismatches (MD=-0.11) and HLA-DR mismatches (MD=-0.13). HLA-B46 antigen (RR=0.39), anti-IL-2-R antibodies induction (RR=0.68), mycophenolate mofetil (RR=0.69), and pretransplant tonsillectomy (RR=0.43) were associated with less IgAN recurrence. Of note, male recipient gender (RR=1.17), related donor (RR=1.53), retransplantation (RR=1.43), hemodialysis (RR=1.68), no induction therapy (RR=1.73), mTOR inhibitor (RR=1.51), angiotensin-converting enzyme inhibitors or angiotensin-receptor blockers (RR=1.63) were risk factors for IgAN recurrence. Recurrence increased the risk of graft loss (RR=2.19).

**Conclusions:**

This study summarized the risk factors for recurrence of IgA nephropathy after kidney transplantation. Well-designed prospective studies are warranted for validation.

**Systematic Review Registration:**

https://www.crd.york.ac.uk/PROSPERO/display_record.php?RecordID=377480, identifier CRD42022377480.

## 1 Introduction

Immunoglobulin A nephropathy (IgAN) is the most prevalent glomerular disease worldwide that can lead to end-stage renal disease (ESRD) with a 10-year renal survival rate ranging from 57% to 91% (1, 2). Kidney transplantation (KT) is the optimal treatment for patients with ESRD. However, there is a risk of IgAN recurrence in renal allografts with a recurrence rate of between 9% and 60%, depending on the time after KT and the IgAN recurrence increased the risk of graft failure (3).

The identification of risk factors for IgAN recurrence is crucial in pre-transplant evaluation, and many studies have been conducted to investigate this issue. However, the results are inconsistent, with some studies indicated that younger age, high human leukocyte-antigen (HLA) matching, and related donor are risk factors, while others found no significant association (4–9). The discrepancy is largely due to the fact that most studies were single-center or had small sample sizes, highlighting the need for further research.

To date, no studies have systematically evaluated the risk factors for IgAN recurrence after KT. Therefore, the present systematic review and meta-analysis aimed to identify risk factors for IgAN recurrence and quantify its impact on clinical outcomes.

## 2 Materials and methods

The systematic review was conducted according to the Preferred Reporting Items for Systematic Reviews and Meta-Analyses 2020 (PRISMA 2020) statement and the Meta-analysis of Observational Studies in Epidemiology (MOOSE) guidelines (10, 11). The protocol was registered in PROSPERO (CRD42022377480).

### 2.1 Search strategy

English or Chinese literature from the following databases were considered: PubMed, EMBASE, Cochrane Library, Scopus, Web of Science, CNKI, Wanfang, CBM, and VIP (from inception to October 3, 2022). We developed a search strategy for each database (**Supplementary Table 1**).

### 2.2 Inclusion and exclusion criteria

Studies were included if they met the following criteria: (1) Patients: those who received a KT due to IgAN induced renal failure; (2) Exposure: various potential risk factors for IgAN recurrence, such as donor and recipient characteristics, primary disease characteristics, immunosuppressive therapy, biomarkers, etc.; (3) Outcome: recurrence of IgAN in kidney allografts; (4) Study design: prospective/retrospective cohort studies or case-control studies. Studies reporting the effect of recurrence on clinical outcomes were also included. The excluded criteria were as follows: (1) Excluded study types: reviews, systematic reviews, case reports, case series, animal studies, comments, conference abstracts, and letters without detailed data, (2) Difficult to differentiate the IgAN recurrence and *de novo* IgAN in allografts, (3) Studies published in languages other than English or Chinese.

### 2.3 Selection of studies and risk bias assessment

Two researchers (YL and YT) independently screened all relevant titles and abstracts of retrieved publications to identify eligible studies. The inclusion and exclusion criteria were then applied to the full text screening. A third reviewer (TS or TL) was consulted to resolve disputes and reach a consensus. The quality and methodological strength of the included studies were assessed by two researchers (YL and YT) using the Newcastle-Ottawa Scale (NOS) (12). Scores of 0-4, 5-6, and 7-9 correspond to poor, moderate, and high quality, respectively.

### 2.4 Data extraction and risk factors identification

Following items were extracted from selected studies: the name of the first author, year of publication, location, research design, demographic characteristics, inclusion and exclusion criteria, follow-up time, and effect of recurrence on outcomes. Number of events and total or associated effect sizes of all reported risk factors were extracted, and those risk factors assessed only by single publication were omitted. Zero counts in a two-by-two table were replaced by 0.5 according to continuity correction. For categorical variables, risk ratios (RRs) and 95% confidence intervals (CIs) were calculated independently by two researchers if reported as number of events and total. Considering that overlapping cohort studies may report different risk factors, the data for each risk factor was screened for overlapping cohort studies and only the studies with the largest sample size were included.

### 2.5 Statistical analysis

For categorical variables, the pooled RRs and 95% CIs were calculated using the Inverse variance method. For continuous variables, mean differences (MDs) or standardized mean differences (SMDs) and 95% CIs were used to pool the differences between the recurrence and non-recurrence groups. The mean and standard deviation were estimated based on data reported as median (interquartile or full range) (13, 14). Heterogeneity across studies was assessed using the I² statistic. If I² > 50%, the heterogeneity is considered to be significant, and the random effects model is used; otherwise, the fixed effect model is used. If the number of studies is greater than 10, Egger’s test is used to evaluate publication bias. All analyses were performed using R software (version 4.2.1).

## 3 Results

### 3.1 Study selection

**Figure 1:**
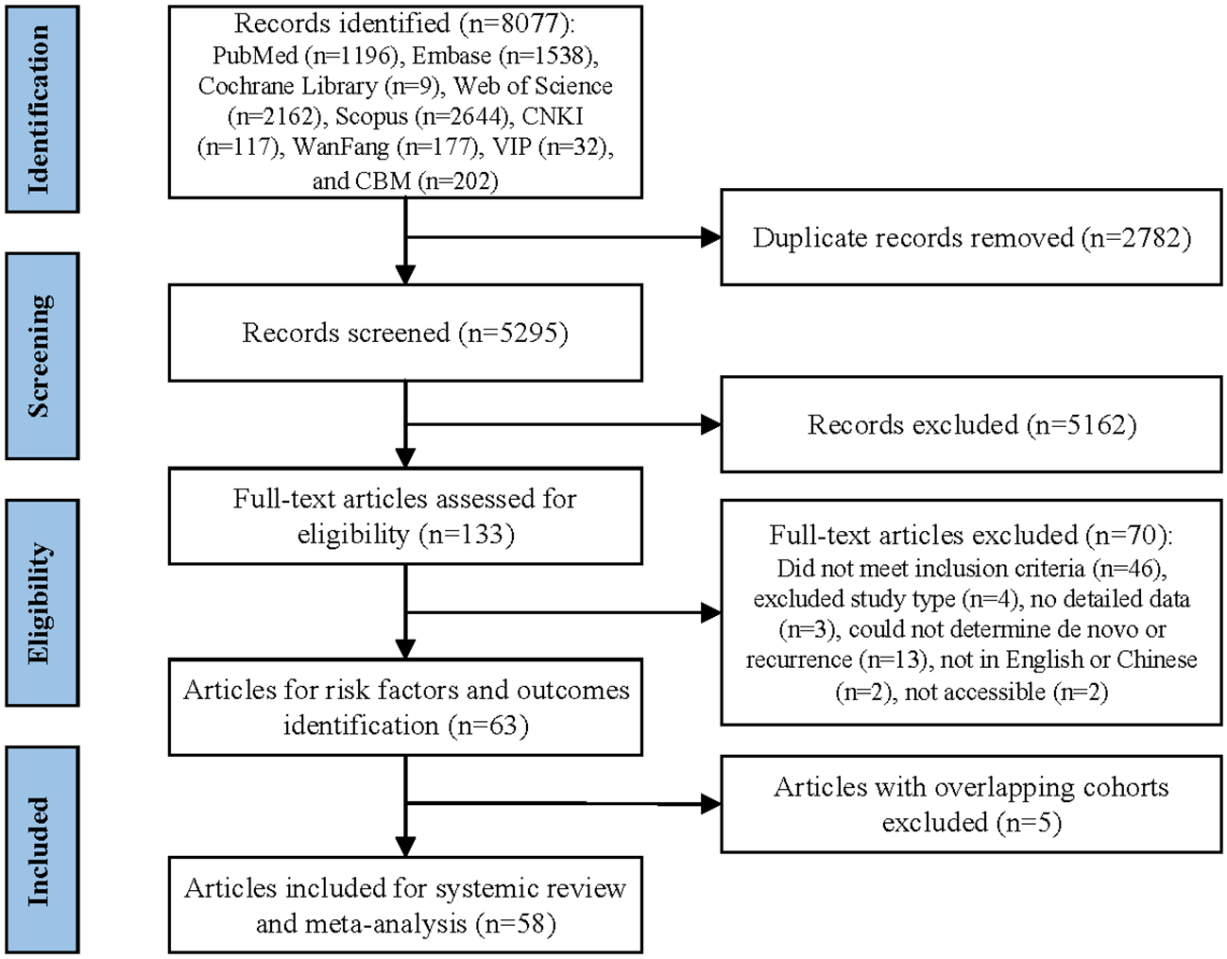
Study selection flowchart.

The study selection process is summarized in **Figure 1**. The initial search retrieved 8077 records, and then 2787 duplicates were removed. After screening the titles and abstracts, 5162 records were excluded and 133 full text articles were assessed for eligibility. Out of these, 58 studies met the inclusion criteria and were included in the systematic review and meta-analysis (4–9, 15–66). The excluded studies and the reasons for their exclusion after full text screening are detailed in **Supplementary Table 2**.

### 3.2 Study characteristics

The characteristics of the included studies are summarized in **Table 1**. The studies were published between 1984 and 2022, with 35 of them being published after 2010. The sample sizes ranged from 13 to 2501. The majority of the studies were retrospective cohort studies (56), while only two were case-control studies. The studies were conducted in various countries, with the majority being from Asia (23), Europe (22), and the USA (7).

**Table 1:** Summary characteristics of included studies.

Study	Study design	Country or region	Study interval	Sample size	Recurrence rate	Female	NOS points
Ahn (2015)	Retrospective cohort	Korea	1989.01-2005.12	56	30.36%	46.43%	6
Allen (2017)	Retrospective cohort	Australia, New Zealand	1985-2014	2501	9.00%	NA	8
Andresdottir (2001)	Retrospective cohort	Netherlands	1969-1997	79	8.86%	NA	6
Avasare (2017)	Retrospective cohort	USA	2001.01-2012.12	62	22.58%	35.48%	7
Bachman (1986)	Retrospective cohort	USA	1969-1984.05	13	46.15%	7.69%	5
Bantis (2008)	Retrospective cohort	Germany	1986-2004	103	15.53%	20.39%	6
Berger (1984)	Retrospective cohort	France	NA	32	53.13%	NA	6
Berthoux (2008)	Retrospective cohort	France	1979.03-2005.12	116	28.45%	NA	6
Berthoux (2015)	Case-control	France	2000-2012	60	NA	40.00%	7
Berthoux (2017)	Retrospective cohort	France	1985.01-2007.12	96	35.42%	17.71%	7
Bjørneklett (2011)	Retrospective cohort	Norway	1988-2004	106	NA	NA	6
Bumgardner (1998)	Retrospective cohort	USA	1980.06-1994.12	61	29.51%	34.43%	8
Chacko (2007)	Retrospective cohort	India	1990.01-2004.05	20	25.00%	25.00%	5
Chandrakantan (2005)	Retrospective cohort	USA	1984.01-2003.08	156	12.82%	NA	6
Choy (2003)	Retrospective cohort	China	1984.01-2001.12	75	18.67%	37.33%	8
Coppo (2007)	Retrospective cohort	Italy	1984-2002.11	61	49.18%	32.79%	7
Courtney (2006)	Retrospective cohort	UK	1977.08-2004.04	75	17.33%	14.67%	6
Di Vico (2018)	Retrospective cohort	Italy	1995.01-2012.12	51	54.90%	NA	7
Freese (1999)	Retrospective cohort	Sweden	1985-1996.12	104	12.50%	20.19%	7
Garnier (2018)	Retrospective cohort	France	2003.01-2013.12	67	20.90%	17.91%	6
Han (2009)	Retrospective cohort	Korea	NA	221	19.91%	42.08%	7
Jäger (2022)	Retrospective cohort	Switzerland	2008.05-2016.12	161	26.71%	17.39%	8
Jeong (2008)	Retrospective cohort	Korea	1992-2003	77	57.14%	27.27%	7
Ji (2016)	Retrospective cohort	China	1996.01-2009.04	148	31.08%	74.32%	5
Jiang (2018)	Retrospective cohort	Australia, New Zealand	1985-2013	2393	9.65%	24.15%	6
Jo (2019)	Retrospective cohort	Korea	2011.1-2015.10	69	15.94%	42.03%	6
Kamal Aziz (2012)	Retrospective cohort	France	1982-2012	142	17.61%	NA	5
Kavanagh (2022)	Retrospective cohort	USA	2005-2019	282	28.37%	30.85%	7
Kawabe (2016)	Retrospective cohort	Japan	1987-2015	21	28.57%	61.90%	6
Kennard (2017)	Retrospective cohort	Australia, New Zealand	1985-2013	2393	NA	NA	6
Kessler (1996)	Retrospective cohort	France	1985.01-1991.06	28	46.43%	14.29%	6
Kim (2001)	Retrospective cohort	Korea	1984.02-1998.10	43	44.19%	NA	7
Kim (2017)	Retrospective cohort	Korea	1990.02-2016.02	95	NA	NA	6
Lee (2019)	Retrospective cohort	Korea	1995.02-2015.03	218	NA	NA	5
Lionaki (2021)	Retrospective cohort	Greece	2000-2018	96	23.96%	29.17%	7
Maixnerova (2021)	Retrospective cohort	Czech	1991-2017	313	14.06%	19.17%	8
Martín‐Penagos (2019)	Retrospective cohort	Spain	1993.01-2015.12	35	40.00%	20.00%	6
McDonald (2006)	Retrospective cohort	Australia, New Zealand	1987.10-2004.12	1386	7.94%	NA	7
Moriyama (2005)	Retrospective cohort	Japan	1992-1999	49	26.53%	34.69%	6
Moroni (2013)	Retrospective cohort	Italy	1981-2010	190	22.11%	21.58%	7
Nakamura (2021)	Retrospective cohort	Japan	NA	15	46.67%	33.33%	5
Namba (2004)	Retrospective cohort	Japan	1980-2001	30	80.00%	33.33%	6
Ng (2007)	Retrospective cohort	Singapore	1984.11-2004.12	29	27.59%	NA	6
Nijim (2016)	Retrospective cohort	USA	1993.04-2014.11	122	18.85%	31.15%	6
Noguchi (2020)	Retrospective cohort	Japan	2002.12-2018.12	135	NA	54.07%	7
Odum (1994)	Retrospective cohort	Australia	1977-1992.09	51	33.33%	6.25%	5
Okumi (2019)	Retrospective cohort	Japan	1995.01-2015.03	299	26.76%	44.15%	7
Ortiz (2012)	Retrospective cohort	Finland, Spain	2001.01-2010.04	65	32.31%	15.38%	8
Park (2021)	Retrospective cohort	Korea	2009-2016	27	48.15%	37.04%	5
Ponticelli (2001)	Retrospective cohort	Italy	1973.07-1999.09	106	32.08%	24.53%	7
Rodas (2020)	Retrospective cohort	Spain	1992-2016	86	26.74%	26.74%	7
Sato (2013)	Retrospective cohort	Japan	1990-2005	184	38.04%	44.02%	6
Sofue (2013)	Retrospective cohort	Japan	2003.08-2011.02	35	34.29%	28.57%	6
Temurhan (2017)	Case-control	Turkey	NA	41	NA	26.83%	6
Uffing (2021)	Retrospective cohort	Europe, North and South America	2005.01-2015.12	504	16.27%	28.17%	9
Von Visger (2014)	Retrospective cohort	USA	1989.06-2008.11	124	21.77%	27.42%	6
Wang (2001)	Retrospective cohort	China	1985.01-1998.12	48	29.17%	54.17%	6
Wang (2021)	Retrospective cohort	China	2008.01-2019.12	149	26.85%	44.30%	6

NOS, Newcastle–Ottawa scale; NA, not available.

### 3.3 Risk of bias

The risk of bias was assessed using the NOS and the results are presented in **Table 1**. The overall quality of the included studies was medium, with 34 studies having a medium quality and 24 having a high quality.

### 3.4 Risk factors for IgAN recurrence

The results of meta-analysis examining risk factors for IgAN recurrence are presented in **Table 2**.

**Table 2:** Meta-analysis of risk factors and outcomes for IgAN recurrence.

Risk factors or outcomes	No. of studies	n	Heterogeneity		Effects model	ES	ES [95% CI]	Z	P	Egger’s test, P
			I²	P						
Recipient										
Age at KT (year)	28	3248	71.60%	< 0.0001	R	MD	-4.27 [-5.76, -2.78]	-5.63	< 0.0001	0.7522
Age at KT (per year)	9	3519	29.30%	0.1848	F	RR	0.96 [0.95, 0.97]	-8.31	< 0.0001	–
Recipient sex (male)	27	3795	6.40%	0.3693	F	RR	1.17 [1.01, 1.35]	2.07	0.0380	0.1139
Recipient body mass index (kg/cm²)	4	677	81.80%	0.0009	R	MD	-1.53 [-3.75, 0.69]	-1.35	0.1762	–
Recipient body mass index (per 1 kg/cm²)	2	722	0.00%	1.0000	F	RR	0.96 [0.92, 1.02]	-1.68	0.0936	–
Donor										
Donor sex (male)	7	983	0.00%	0.5525	F	RR	0.90 [0.73, 1.11]	-1.00	0.3171	–
Donor age at KT (year)	15	1956	34.70%	0.0910	F	MD	-2.19 [-3.46, -0.93]	-3.40	0.0007	0.4067
Donor age at KT (per year)	3	389	0.00%	0.4354	F	RR	0.99 [0.97, 1.01]	-0.79	0.4314	–
Living donor	26	4472	48.80%	0.0029	F	RR	1.02 [0.90, 1.14]	0.28	0.7781	0.0003
Related donor	21	2559	62.90%	< 0.0001	R	RR	1.53 [1.24, 1.88]	4.02	< 0.0001	0.0001
Living related donor (vs. living unrelated donor)	10	1221	21.40%	0.2461	F	RR	1.69 [1.31, 2.18]	3.99	< 0.0001	0.1001
Primary disease										
Age at IgAN diagnosis (year)	6	909	71.70%	0.0034	R	MD	-2.52 [-6.73, 1.69]	-1.17	0.2412	–
Age at IgAN diagnosis (per year)	2	417	86.00%	0.0075	R	RR	0.99 [0.93, 1.05]	-0.43	0.6699	–
Time from IgAN diagnosis to ESRD (year)	13	1448	0.00%	0.6548	F	MD	-1.84 [-2.43, -1.25]	-6.14	< 0.0001	0.8399
Time from IgAN diagnosis to ESRD (per year)	3	293	0.00%	0.4097	F	RR	0.94 [0.91, 0.97]	-3.41	0.0007	–
History of KT										
History of KT	12	4421	47.40%	0.0342	F	RR	1.43 [1.24, 1.65]	4.98	< 0.0001	0.5062
Dialysis history										
Time on dialysis (month)	19	2164	44.60%	0.0193	F	MD	-3.14 [-4.18, -2.09]	-5.88	< 0.0001	0.5114
Hemodialysis	3	689	1.70%	0.3615	F	RR	1.68 [1.04, 2.71]	2.13	0.0331	–
Pre-emptive transplant	10	1569	52.70%	0.0249	R	RR	1.12 [0.77, 1.62]	0.60	0.5501	0.5854
Comorbidities										–
Hypertension	7	600	44.80%	0.0923	F	RR	1.09 [0.76, 1.57]	0.49	0.6245	–
Diabetes	3	276	0.00%	0.6065	F	RR	1.22 [0.73, 2.03]	0.75	0.4547	–
Histocompatibility										
Panel reactive antibodies	4	689	65.90%	0.0320	R	MD	0.95 [-3.55, 5.45]	0.41	0.6795	–
Panel reactive antibodies >50%	3	543	0.00%	0.5899	F	RR	0.66 [0.34, 1.29]	-1.21	0.2259	–
Pretransplant donor specific antibody	5	1267	67.70%	0.0148	R	RR	1.01 [0.53, 1.94]	0.04	0.9650	–
ABO-incompatibility	4	505	0.00%	0.8607	F	RR	0.89 [0.60, 1.31]	-0.60	0.5487	–
HLA-A/B/DR mismatches	19	2414	38.40%	0.0455	F	MD	-0.11 [-0.22, -0.00]	-2.00	0.0455	0.6727
HLA-A/B mismatches	2	251	0.00%	0.7481	F	MD	0.09 [-0.05, 0.22]	1.20	0.2294	–
HLA-DR mismatches	2	251	0.00%	0.7494	F	MD	-0.13 [-0.22, -0.05]	-3.00	0.0027	–
HLA-A/B/DR full match	4	905	31.3%	0.2244	F	RR	1.88 [1.14, 3.11]	2.47	0.0135	–
HLA-A full match	2	98	0.00%	0.8560	F	RR	1.48 [0.70, 3.09]	1.03	0.3021	–
HLA-B full match	4	180	36.70%	0.1921	F	RR	1.12 [0.72, 1.76]	0.51	0.6084	–
HLA-DR full match	4	229	85.80%	< 0.0001	R	RR	0.71 [0.15, 3.38]	-0.43	0.6693	–
HLA identied related donor (vs. other)	2	152	78.70%	0.0301	R	RR	2.91 [0.7, 12.01]	1.47	0.1406	–
HLA identied related donor (vs. not identied related donor)	3	92	18.50%	0.2932	F	RR	1.14 [0.70, 1.88]	0.53	0.5964	–
HLA-A2	7	661	36.90%	0.1471	F	RR	1.03 [0.78, 1.37]	0.21	0.8300	–
HLA-B35	8	1051	0.00%	0.7456	F	RR	1.25 [0.95, 1.64]	1.59	0.1116	–
HLA-B46	3	290	0.00%	0.5821	F	RR	0.39 [0.16, 0.95]	-2.06	0.0392	–
HLA-DR3	2	466	0.00%	0.9142	F	RR	1.14 [0.84, 1.55]	0.86	0.3883	–
HLA-DR4	4	397	82.80%	0.0006	R	RR	2.91 [0.94, 8.97]	1.86	0.0630	–
Induction therapy										
None	9	1453	70.50%	0.0007	R	RR	1.73 [1.16, 2.58]	2.69	0.0071	–
Anti-IL-2-R antibodies	14	2102	79.80%	< 0.0001	R	RR	0.68 [0.47, 0.99]	-2.02	0.0429	0.8046
Antithymocyte globulin	13	1844	74.10%	< 0.0001	R	RR	0.97 [0.64, 1.47]	-0.15	0.8784	0.0189
Anti-CD20 antibodies	2	312	74.20%	0.0488	R	RR	0.63 [0.22, 1.82]	-0.85	0.3951	–
Maintenance therapy										
Tacrolimus	16	2146	52.20%	0.0077	R	RR	0.90 [0.68, 1.19]	-0.72	0.4741	0.5163
Cyclosporine	15	1701	41.90%	0.0445	F	RR	1.07 [0.90, 1.28]	0.77	0.4414	0.2776
MMF	12	1846	24.90%	0.1996	F	RR	0.69 [0.56, 0.86]	-3.28	0.0010	0.9129
Azathioprine	6	684	2.00%	0.4036	F	RR	1.18 [0.85, 1.64]	0.99	0.3239	–
mTOR inhibitor	7	602	0.00%	0.5686	F	RR	1.51 [1.10, 2.06]	2.57	0.0102	–
Steroids	10	3504	74.1%	< 0.0001	R	RR	0.88 [0.56, 1.38]	-0.55	0.5831	0.3347
Graft rejection										
Rejection	8	1305	36.00%	0.1414	F	RR	1.10 [0.83, 1.46]	0.69	0.4932	–
T cell-mediated rejection	2	361	34.80%	0.2155	F	RR	1.42 [0.96, 2.09]	1.75	0.0800	–
Antibody-mediated rejection	3	374	0.00%	0.9932	F	RR	0.85 [0.52, 1.38]	-0.67	0.5042	–
Acute rejection	13	2063	0.00%	0.6052	F	RR	1.13 [0.96, 1.34]	1.43	0.1532	0.9843
Acute T cell-mediated rejection	2	754	0.00%	0.6059	F	RR	1.39 [0.99, 1.94]	1.92	0.0547	–
Acute antibody-mediated rejection	2	754	81.70%	0.0195	R	RR	1.25 [0.35, 4.47]	0.35	0.7279	–
Chronic rejection	3	468	28.60%	0.2465	F	RR	1.33 [0.91, 1.93]	1.47	0.1403	–
Other therapy										
ACEI/ARB	8	640	0.00%	0.6797	F	RR	1.80 [1.42, 2.28]	4.87	< 0.0001	–
Plasma exchange	2	312	0.00%	0.4185	F	RR	0.80 [0.54, 1.18]	-1.12	0.2639	–
Tonsillectomy	2	350	0.00%	0.6420	F	RR	0.43 [0.23, 0.79]	-2.69	0.0072	–
Steroids use before KT	2	173	0.00%	0.3936	F	RR	0.97 [0.58, 1.62]	-0.13	0.8995	–
Immunosuppression use before KT	3	279	62.00%	0.0718	R	RR	1.27 [0.60, 2.71]	0.62	0.5363	–
Other										
Cold ischemia time (hour)	7	1356	12.90%	0.3312	F	MD	0.10 [-0.76, 0.96]	0.23	0.8188	–
Delayed graft function	5	443	18.60%	0.2960	F	RR	0.70 [0.43, 1.12]	-1.48	0.1377	–
Cytomegalovirus infection	2	476	0.00%	0.8245	F	RR	0.79 [0.45, 1.38]	-0.84	0.4023	–
BK virus infection	2	517	0.00%	0.5896	F	RR	1.32 [0.67, 2.57]	0.81	0.4205	–
Outcomes										
Graft loss	30	5986	82.80%	< 0.0001	R	RR	2.19 [1.60, 3.01]	4.88	< 0.0001	0.0040
Death	4	703	0.00%	0.5712	F	RR	0.77 [0.42, 1.41]	-0.85	0.3952	–
Infection	2	339	0.00%	0.8135	F	RR	1.02 [0.66, 1.59]	0.10	0.9231	–

IgAN, immunoglobulin A nephropathy; ES, effect size; CI, confidence interval; KT, kidney transplantation; R, random effects model; F, fixed effect model; MD, mean difference; RR, risk ratio; ESRD, end stage renal disease; HLA, human leukocyte antigen; MMF, mycophenolate mofetil; ACEI, angiotensin-converting enzyme inhibitors; ARB, angiotensin receptor blockers.

#### 3.4.1 Demographic characteristics of recipients and donors

The study found that recipients with recurrent IgAN were younger at KT than those without recurrence (MD = -4.27 years, 95% CI: -5.76 to -2.78, I² = 71.60%). The pooled RR showed that the risk of recurrence decreased by 4% for each increase in age of 1 year at KT (RR = 0.96, 95%CI: 0.95-0.97, I² = 29.30%). The donor age was also found to be younger in the recurrent group (MD = -2.19 years, 95% CI: -3.46 to -0.93, I² = 34.70%), but the pooled RR of 389 participants from 3 studies was not significant (RR = 0.99, 95%CI: 0.97 to 1.01, I² = 0.00%). Male recipients had a 17% increased risk of recurrence compared to female recipients (RR = 1.17, 95%CI: 1.01 to 1.35, I² = 6.40%). There were no significant differences between the recurrence and non-recurrence groups with respect to recipient body mass index or donor sex.

#### 3.4.2 Donor type

There was no difference in the risk of recurrence among recipients with living donors compared to those with deceased donors (RR = 1.02, 95% CI: 0.90 to 1.14, I² = 48.80%). Recipients with related donors had a higher risk of recurrence compared to those with unrelated donors (RR = 1.53, 95% CI: 1.24 to 1.88, I² = 62.90%). Further analysis found that recipients with living related donors had a higher risk of recurrence compared with those with living unrelated donors (RR = 1.69, 95% CI: 1.31 to 2.18, I² = 21.40%).

#### 3.4.3 Primary disease

The age at diagnosis of IgAN did not differ between the recurrence and non-recurrence groups. However, the time from IgAN diagnosis to ESRD was shorter in the recurrence group compared to the non-recurrence group, with an MD of -1.84 years (95% CI: -2.43 to -1.25, I² = 0.00%). The RR value for the time from diagnosis to ESRD showed each additional year was associated with a 6% reduction in recurrence (RR = 0.94, 95% CI: 0.91 to 0.97, I² = 0.00%). This suggests that patients with a faster progression of primary disease were more susceptible to recur.

#### 3.4.4 Dialysis history

Recurrent group had short dialysis duration (MD = -3.14 months, 95% CI: -4.18 to -2.09, I² = 44.60%) and hemodialysis (RR = 1.68, 95% CI: 1.04 to 2.71, I² = 1.70%) were identified as risk factors for recurrence, while pre-emptive transplantation was not found to be a significant risk factor.

#### 3.4.5 Histocompatibility features

In patients with recurrent IgAN, lower total HLA mismatches (MD = -0.11, 95% CI: -0.22 to -0.00, I² = 38.40%) and lower HLA-DR mismatches (MD = -0.13, 95% CI: -0.22 to -0.05, I² = 0.00%) were observed, but no significant difference was found in HLA-A and B. The pooled RR showed an increased risk of recurrence in recipients with HLA full match (RR = 1.88, 95% CI: 1.14 to 3.11, I² = 31.3%). However, compared with more than 1 mismatch, HLA-A, B, or DR full match were all found to have no effect on recurrence. For specific HLA antigens, HLA-B46 reduced the risk of recurrence (RR = 0.39, 95% CI: 0.16 to 0.95, I² = 0.00%), while HLA-A2, HLA-B35 HLA-DR3, and HLA-DR4 had no effect. ABO incompatibility, donor-specific antibodies, and panel reactive antibodies did not affect recurrence.

#### 3.4.6 Immunosuppressive therapy

Patients without induction therapy had an increased risk of recurrence (RR = 1.73, 95% CI: 1.16 to 2.58, I² = 70.50%). The use of anti-IL-2-R antibodies was found to reduce the risk of recurrence by 32% (RR = 0.68, 95% CI: 0.47 to 0.99, I² = 79.80%), while antithymocyte globulin and anti-CD20 antibodies had no effect. For maintenance agents, mycophenolate mofetil (MMF) reduced the risk of recurrence (RR = 0.69, 95% CI: 0.56 to 0.86, I² = 24.90%) and use of mTOR inhibitor is associated with a higher risk (RR = 1.51, 95% CI: 1.10 to 2.06, I² = 0.00%), while steroids, tacrolimus, cyclosporine, and azathioprine had no significant effect. Pretransplant steroids or immunosuppressant exposure did not affect recurrence.

#### 3.4.7 Other therapy

Recipients with recurrent IgAN were more likely to use angiotensin-converting enzyme inhibitors (ACEIs) or angiotensin-receptor blockers (ARBs) (RR = 1.63, 95%CI: 1.30 to 2.05, I² = 0.00%). Patients who had tonsillectomy before KT had a 57% lower risk of recurrence (RR = 0.43, 95% CI: 0.23 to 0.79, I² = 0.00%). Plasma exchange had no effect on recurrence.

#### 3.4.8 Other factors

Recipients with KT history had a 43% increased risk of recurrence compared to those with first KT (RR = 1.43, 95% CI: 1.24 to 1.65, I² = 47.40%). Factors such as graft rejection, hypertension, diabetes, cold ischemia time, delayed graft function, cytomegalovirus or BK virus infection, did not have an impact on recurrence.

#### 3.4.9 Serum biomarkers

Our findings indicated that serum immunoglobulin G autoantibodies (IgG), IgA, and galactose-deficient IgA1 (Gd-IgA1) levels for recurrence were not predictive of IgAN recurrence (**Table 3**).

**Table 3:** Meta-analysis of laboratory data for IgAN recurence.

Laboratory parameters	No. of studies	n	Heterogeneity		Effects model	ES	ES [95% CI]	Z	P
			I²	P					
IgG (g/L)									
Baseline	3	268	79.70%	0.0073	R	MD	1.48 [-0.19, 3.16]	1.74	0.0827
IgA (mg/L)									
Baseline	5	615	83.20%	< 0.0001	R	MD	0.48 [-0.10, 1.05]	1.63	0.1033
6 month	2	225	88.40%	0.0033	R	MD	0.19 [-0.83, 1.20]	0.36	0.7197
1 year	3	561	81.30%	0.0048	R	MD	0.32 [-0.28, 0.92]	1.04	0.2997
Gd-IgA1									
Baseline	5	400	2.70%	0.3911	F	SMD	0.05 [-0.17, 0.27]	0.44	0.6634
Hematuria									
1 year	3	232	89.50%	0.0021	R	SMD	1.29 [0.10, 2.47]	2.13	0.0328
3 year	3	232	97.90%	< 0.0001	R	SMD	1.94 [-0.65, 4.54]	1.47	0.1425
5 year	2	183	99.30%	< 0.0001	R	SMD	5.09 [-3.10, 13.28]	1.22	0.2231
Post KT *	6	646	77.20%	0.0005	R	RR	3.27 [1.63, 6.55]	3.33	0.0009
Proteinuria									
6 month	2	173	67.50%	0.0795	R	SMD	0.33 [-0.31, 0.96]	1.01	0.3128
1 year	5	477	33.40%	0.1989	F	SMD	0.37 [0.17, 0.58]	3.52	0.0004
3 year	2	197	0.00%	0.9672	F	SMD	1.76 [1.41, 2.11]	9.78	< 0.0001
5 year *	2	355	58.50%	0.1208	R	RR	2.19 [1.12, 4.31]	2.28	0.0227
Time at biopsy	4	182	0.00%	0.6860	F	SMD	0.22 [-0.08, 0.53]	1.46	0.1431
Time at biopsy *	2	334	74.90%	0.0461	R	RR	3.91 [0.88, 17.25]	1.80	0.0722
Last follow up	7	587	85.30%	< 0.0001	R	SMD	1.25 [0.55, 1.94]	3.49	0.0005
eGFR (ml/min/1.73m^2^)									
Baseline	2	197	0.00%	0.8518	F	MD	-0.05 [-2.28, 2.18]	-0.05	0.9626
6 month	5	708	29.70%	0.2233	F	MD	0.05 [-2.23, 2.33]	0.04	0.966
2 year	2	307	95.10%	< 0.0001	R	MD	-14.37 [-37.20, 8.46]	-1.23	0.2173
3 year	3	608	98.90%	< 0.0001	R	MD	-11.31 [-30.18, 7.57]	-1.17	0.2403
5 year	3	608	99.30%	< 0.0001	R	MD	12.87 [-38.14, 12.40]	-1.00	0.3181
Last follow up	4	410	0.00%	0.9340	F	MD	-12.37 [-17.25, -7.49]	-4.97	< 0.0001
Serum creatinine (mg/dL)									
Baseline	4	358	83.80%	0.0003	R	MD	-0.05 [-0.18, 0.08]	-0.76	0.4490
6 month	2	173	92.30%	0.0003	R	MD	-0.23 [-0.78, 0.33]	-0.80	0.4240
1 year	7	469	0.00%	0.7372	F	MD	-0.10 [-0.30, 0.10]	-1.00	0.3186
3 year	3	253	89.30%	< 0.0001	R	MD	0.58 [-0.14, 1.30]	1.57	0.1156
5 year	4	307	99.80%	< 0.0001	R	MD	1.01 [-0.55, 2.57]	1.27	0.2050
Last follow up	8	562	37.90%	0.1269	F	MD	0.49 [0.35, 0.64]	6.60	< 0.0001

IgAN, immunoglobulin A nephropathy; ES, effect size; CI, confidence interval; R, random effects model; F, fixed effect model; MD, mean difference; SMD, standardized mean difference; RR, risk ratio; IgG, immunoglobulin G; IgA, immunoglobulin A; Gd-IgA1, galactose deficiency IgA1; KT, kidney transplantation; eGFR, estimated glomerular filtration rate.

*dichotomous variables.

### 3.5 Meta-analysis of hematuria and proteinuria in recurrent IgAN

The level of hematuria was found to be higher in patients with recurrence one year after transplantation, compared to those without recurrence (SMD = 1.29, 95% CI: 0.10 to 2.47, I² = 89.50%). Additionally, patients with recurrence were more likely to have hematuria, regardless of the time of occurrence (RR = 3.27, 95% CI: 1.63 to 6.55, I² = 77.20%). However, there was no significant difference in hematuria levels between the 3-year and 5-year follow-up periods. Urinary protein levels were also higher in patients with recurrence, and this difference was significant at follow-up periods (1 year, 3 years, 5 years, and the final follow-up).

### 3.6 Clinical outcomes of IgAN recurrence

A total of 27 studies were analyzed to determine the RR for graft loss in patients with IgAN recurrence. The results showed that the presence of IgAN recurrence was associated with poorer graft survival (RR = 2.19, 95% CI: 1.60 to 3.01, I² = 82.80%) (**Table 2**). IgAN recurrence did not have impact on post-transplant death or infection rates. With respect to renal function, no significant difference was found during the other time periods, except for worse renal function in patients with recurrence at the final follow-up (estimated glomerular filtration rate [eGFR]: MD = -12.37 ml/min/1.73m^2^, 95% CI: -17.25 to -7.49, I² = 0.00%; serum creatinine: MD = 0.49 mg/dL, 95% CI: 0.35 to 0.64, I² = 37.90%) (**Table 3**).

### 3.7 Publication bias

The majority of the P values of Egger’s test were not significant, indicating that there was no significant publication bias (**Table 2**). However, it is important to note that publication bias may be present in the analysis of the pooled RRs for donor types (living donor: p = 0.0003; related donor: p = 0.0001), induction with antithymocyte globulin (p = 0.0189) and graft loss outcome (p = 0.0040).

## 4 Discussion

In the present systematic review and meta-analysis, our aim was to evaluate the risk factors associated with the recurrence of IgAN and provide a comprehensive summary of the outcomes based on relevant articles published until now. The immediate consequence of IgAN recurrence is an increased risk of graft loss, and similar to primary IgAN, treatment options for recurrent IgAN are limited. Thus, by studying the risk factors and employing appropriate risk stratification and preventive measures, it is possible to determine the likelihood of IgAN recurrence at an early stage and consequently reduce the recurrence rate. Although some previous studies have explored the risk factors for IgAN recurrence, their findings have been controversial due to variations in selection criteria, sample size, and study design. Therefore, in this meta-analysis, we aim to identify potential risk factors for IgAN recurrence, with key findings summarized in **Table 4**.

**Table 4:** Summary of risk factors for IgAN recurrence.

Risk factors	Protective factors
Young recipient	HLA-B46 antigen
Young donor	Induction with anti-IL-2-R antibodies
Rapid progression from IgAN diagnosis to ESKD	MMF
Short time on dialysis	Pretransplant tonsillectomy
Low total HLA mismatches	
Low HLA-DR mismatches	
Male recipient	
Related donor	
Retransplantation	
Hemodialysis	
No induction therapy	
mTOR inhibitor	
ACEI/ARB	

IgAN, immunoglobulin A nephropathy; ESRD, end stage renal disease; HLA, human leukocyte antigen; ACEI, angiotensin-converting enzyme inhibitors; ARB, angiotensin receptor blockers; MMF, mycophenolate mofetil.

IgAN is a systemic autoimmune disease which affects both the native and allograft kidney with a high recurrence (67). The “multi-hit” hypothesis is widely accepted as the pathogenesis of IgAN (67, 68). Although this hypothesis is yet to be proven, it has gained wide acceptance due to the available evidence (67). Therefore, we have attempted to establish a connection between our findings and the four stages of its pathogenesis.

The first step in the pathogenesis is an increase in circulating abnormal IgA (Gd-IgA1). The serum level of Gd-IgA1 was found to be elevated in patients with native IgAN (69). Additionally, patients who underwent KT also exhibited higher serum levels of Gd-IgA1 compared to healthy controls, both at diagnosis and at transplant (26). However, our pooled results indicate that serum Gd-IgA1 levels did not serve as a predictor for recurrence, but it is important to note that these measurements were taken at baseline. To the best of our knowledge, there have been no studies investigating the relationship between dynamic changes in serum Gd-IgA1 levels post-KT and the occurrence of recurrence. Further research in this area is imperative. The immune cells responsible for the production of Gd-IgA1 are present in the mucosa-associated lymphoid tissues, with the tonsil being a key component of these tissues (70, 71). Currently, the KDIGO clinical practice guidelines do not recommend tonsillectomy as a part of the treatment for native IgAN. However, studies have shown that tonsillectomy can lead to clinical remission and lower the incidence of ESRD in patients with native IgAN (72, 73). This finding has also been supported by a recent study with a large sample size (74). In the case of KT, our pooled results suggest that tonsillectomy may also prevent recurrence, though this conclusion is based on only 350 transplants from two studies (5, 34). Another aspect to consider is the associated risk of complications from tonsillectomy, which has been reported to range from 2.8% to 3.2% (74, 75). Therefore, it is necessary to conduct well-designed prospective studies to thoroughly evaluate the benefits and risks of tonsillectomy in the prevention and treatment of recurrent IgAN within the context of KT.

Anti-glycan immunoglobulin G autoantibodies (IgG) bind to abnormal IgA to form circulating immune complexes (76). However, no predictive effect of serum IgG on recurrence was detected in present study. Serum IgG antiglycan autoantibody level at transplant has been found to predict recurrence, but this was investigated in only one study, limiting further synthetic analysis (26). Genetic background, particularly major histocompatibility complex (MHC) sites, also play important roles in native IgAN disease (77–79). However, these antigens have not been systematically studied in the context of recurrent IgAN, and our results suggest that HLA-B46 is a protective factor. We found that patients with rapid progression of the primary disease were more likely to recur, possibly due to a stronger ongoing systemic autoimmune response after KT. Additionally, our study found that older recipients had a lower risk of recurrence, which may be due to a decrease in the production of autoantibodies by the immune system with age, consistent with what is observed in primary IgAN. Theoretically, regulation of the pathogenic immune pathway may alter the natural course of the disease. However, the role of immunosuppressive drugs in native IgAN remains controversial. Patients not receiving induction therapy had a 73% increased risk of recurrence, but this may be related to HLA matching, as patients with lower HLA mismatch were more likely not to receive induction therapy. The soluble IL-2 receptor α has been found to be associated with the progression of native IgAN (80). The anti-IL-2-R antibody targets the CD25 antigen (IL-2-R) on activated T lymphocytes, thereby blocking IL-2 binding (81). As a result, there is a cell cycle arrest in the G0 or G1 phase, which inhibits T cell proliferation. This suggests that the anti-IL-2-R antibody may inhibit the production of autoantibodies mediated by the above-mentioned pathway and therefore prevent the recurrence of IgAN after KT. Of the maintenance drugs, MMF was found to lower the risk of recurrence, possibly due to its ability to inhibit B and T lymphocyte proliferation and reduce the production of autoantibodies and Gd-IgA1 (82). Although mTOR inhibitors have also been associated with inhibition of T cell proliferation, their use was found to be associated with an increased risk of recurrence, possibly because mTOR inhibitors increase proteinuria and thus increase the chance of biopsy, leading to the detection of subclinical pathological recurrence findings (83, 84).

In the final stage of pathogenesis, mesangial deposition of circulating Gd-IgA1-antiglycan IgG immune complex in the renal allograft results in cell activation and glomerular injury, ultimately leads to recurrence. Transferrin receptor 1 (TfR1) was identified as an IgA1 receptor expressed on human mesangial cells and has affinity with the immune complex formed by Gd-IgA1 and IgG (85). Our observation that related donors and patients with low HLA mismatch are more likely to recur may indicate that allografts with similar genetic backgrounds may express more IgA1 receptors and therefore have higher affinity for host circulating immune complexes. Glomerular injury results in hematuria and proteinuria production, as our study found that patients had higher levels of urinary red blood cells and urinary protein before and after recurrence, which reminds us that more attention should be paid to urinalysis in the follow-up of these patients to determine the timing of biopsy. The role of ACEI/ARB in conservative therapy for native or recurrent IgAN is well established (1, 86). Our study found that recurrent patients were more likely to receive ACEI/ARB therapy, possibly because patients with recurrence were more prone to proteinuria during the course of the disease, leading to confounding bias. Furthermore, other medications, such as MMF and other immunosuppressants may conceal the protective effect of ACEI/ARB in KT (82).

Other risk factors were also identified in our study. Male recipients were more likely to recur, consistent with the higher prevalence observed in males in native IgAN (68). The donor age of recurrent recipients was also found to be younger. The duration of dialysis in recurrent patients was 3.14 months shorter than in non-recurrent patients. It is worth noting that the duration of dialysis in living related donor recipients is usually short, and we observed that the recurrence risk of these individuals is lower. However, preemptive transplant patients and patients receiving dialysis did not differ in recurrence risk. Since recurrence increases the risk of graft failure, and the same risk factors make it easier to recur after a second or subsequent transplant. Our study lacked analysis of immunopathological and histopathological risk factors. Previous studies have shown that complement deposition, such as C4d in the glomeruli, plays an important role in graft loss in recurrent IgAN (87). Given the low prevalence of procedural biopsy, it is difficult to analyze its predictive effect on the risk of recurrence.

This study has several limitations. All the studies included in this meta-analysis are retrospective studies, which may introduce inevitable biases. Furthermore, the overall quality of the studies is rated as medium. All the patients included in the analysis had a biopsy-confirmed diagnosis of IgAN, which could potentially result in selection bias and underestimate the recurrent incidence of IgAN. Additionally, some of the analysis is based on univariate data, which could be influenced by confounding factors. A number of the identified risk factors are based on limited studies and small sample sizes, so further research is required to verify these findings. Moreover, most of the results from Egger’s test showed p-values greater than 0.05, indicating no significant publication bias in the included literature. However, we were unable to analyze publication bias for the analysis with less than 10 included articles. This limitation arises from the fact that the power of Egger’s test greatly decreases when the number of studies is small. Additionally, we restricted the study to articles published in English and Chinese, potentially excluding studies in other relevant languages and introducing publication bias. As a result, it is advisable to exercise caution when interpreting the results of this meta-analysis.

In conclusion, this meta-analysis has identified several factors associated with IgAN recurrence after KT. As patients with recurrence had poor graft outcomes, our findings may improve pre-transplant evaluation of individuals with IgAN induced renal failure. By properly stratifying risk and implementing appropriate interventions, it might be of help to enhance long-term outcomes in this population.

## Data availability statement

The original contributions presented in the study are included in the article/**Supplementary Material**. Further inquiries can be directed to the corresponding author.

## Author contributions

YL: data curation, formal analysis, investigation, methodology, software, visualization, writing – original draft. TY: writing – original draft, data curation, formal analysis, investigation. TL: funding acquisition, supervision, validation, writing – review & editing. TS: supervision, validation, writing – review & editing, conceptualization, funding acquisition, project administration, software.
