# Supplementary material for: Risk factors and outcomes of IgA nephropathy recurrence after kidney transplantation: a systematic review and meta-analysis
Source: Front Immunol. 2023 Nov 28;14:1277017. doi: 10.3389/fimmu.2023.1277017 (PMC10713786; doi:10.3389/fimmu.2023.1277017)
Supplement: Supplementary file 1 [file Table_1.docx]

Risk factors and outcomes of IgA nephropathy recurrence after kidney transplantation: a systematic review and meta-analysis

Supplementary Material

**Table of contents**

**Supplementary Table 12**

**Supplementary Table 25**

**References8**

**Supplementary Table 1.** Search strategy

| **Database** | **Search methods** |
| --- | --- |
| **PubMed** | ("glomerulonephritis, iga"[MeSH Terms] OR ("glomerulonephritis, iga"[MeSH Terms] OR ("glomerulonephritis"[All Fields] AND "iga"[All Fields]) OR "iga glomerulonephritis"[All Fields] OR ("glomerulonephritides"[All Fields] AND "iga"[All Fields]) OR "glomerulonephritides iga"[All Fields] OR ("glomerulonephritis, iga"[MeSH Terms] OR ("glomerulonephritis"[All Fields] AND "iga"[All Fields]) OR "iga glomerulonephritis"[All Fields] OR ("berger s"[All Fields] AND "disease"[All Fields]) OR "berger s disease"[All Fields]) OR ("glomerulonephritis, iga"[MeSH Terms] OR ("glomerulonephritis"[All Fields] AND "iga"[All Fields]) OR "iga glomerulonephritis"[All Fields] OR ("bergers"[All Fields] AND "disease"[All Fields])) OR ("glomerulonephritis, iga"[MeSH Terms] OR ("glomerulonephritis"[All Fields] AND "iga"[All Fields]) OR "iga glomerulonephritis"[All Fields] OR ("iga"[All Fields] AND "glomerulonephritis"[All Fields])) OR ("glomerulonephritis, iga"[MeSH Terms] OR ("glomerulonephritis"[All Fields] AND "iga"[All Fields]) OR "iga glomerulonephritis"[All Fields] OR ("nephropathy"[All Fields] AND "iga"[All Fields]) OR "nephropathy iga"[All Fields]) OR ("glomerulonephritis, iga"[MeSH Terms] OR ("glomerulonephritis"[All Fields] AND "iga"[All Fields]) OR "iga glomerulonephritis"[All Fields] OR "iga nephropathy 1"[All Fields]) OR ("glomerulonephritis, iga"[MeSH Terms] OR ("glomerulonephritis"[All Fields] AND "iga"[All Fields]) OR "iga glomerulonephritis"[All Fields] OR "nephropathy 1 iga"[All Fields]) OR ("glomerulonephritis, iga"[MeSH Terms] OR ("glomerulonephritis"[All Fields] AND "iga"[All Fields]) OR "iga glomerulonephritis"[All Fields] OR "immunoglobulin a nephropathy"[All Fields]) OR ("glomerulonephritis, iga"[MeSH Terms] OR ("glomerulonephritis"[All Fields] AND "iga"[All Fields]) OR "iga glomerulonephritis"[All Fields] OR "nephropathy immunoglobulin a"[All Fields]) OR ("glomerulonephritis, iga"[MeSH Terms] OR ("glomerulonephritis"[All Fields] AND "iga"[All Fields]) OR "iga glomerulonephritis"[All Fields] OR ("nephritis"[All Fields] AND "iga"[All Fields] AND "type"[All Fields])) OR ("glomerulonephritis, iga"[MeSH Terms] OR ("glomerulonephritis"[All Fields] AND "iga"[All Fields]) OR "iga glomerulonephritis"[All Fields] OR ("iga"[All Fields] AND "type"[All Fields] AND "nephritis"[All Fields])) OR ("glomerulonephritis, iga"[MeSH Terms] OR ("glomerulonephritis"[All Fields] AND "iga"[All Fields]) OR "iga glomerulonephritis"[All Fields] OR ("berger"[All Fields] AND "disease"[All Fields]) OR "berger disease"[All Fields]) OR ("glomerulonephritis, iga"[MeSH Terms] OR ("glomerulonephritis"[All Fields] AND "iga"[All Fields]) OR "iga glomerulonephritis"[All Fields] OR ("iga"[All Fields] AND "nephropathy"[All Fields]) OR "iga nephropathy"[All Fields]))) AND ("Kidney Transplantation"[MeSH Terms] OR ("Kidney Transplantation"[MeSH Terms] OR ("kidney"[All Fields] AND "transplantation"[All Fields]) OR "Kidney Transplantation"[All Fields] OR ("renal"[All Fields] AND "transplantation"[All Fields]) OR "renal transplantation"[All Fields] OR ("Kidney Transplantation"[MeSH Terms] OR ("kidney"[All Fields] AND "transplantation"[All Fields]) OR "Kidney Transplantation"[All Fields] OR ("renal"[All Fields] AND "transplantations"[All Fields]) OR "renal transplantations"[All Fields]) OR ("Kidney Transplantation"[MeSH Terms] OR ("kidney"[All Fields] AND "transplantation"[All Fields]) OR "Kidney Transplantation"[All Fields] OR ("transplantations"[All Fields] AND "renal"[All Fields]) OR "transplantations renal"[All Fields]) OR ("Kidney Transplantation"[MeSH Terms] OR ("kidney"[All Fields] AND "transplantation"[All Fields]) OR "Kidney Transplantation"[All Fields] OR ("transplantation"[All Fields] AND "renal"[All Fields]) OR "transplantation renal"[All Fields]) OR ("Kidney Transplantation"[MeSH Terms] OR ("kidney"[All Fields] AND "transplantation"[All Fields]) OR "Kidney Transplantation"[All Fields] OR ("grafting"[All Fields] AND "kidney"[All Fields]) OR "grafting kidney"[All Fields]) OR ("Kidney Transplantation"[MeSH Terms] OR ("kidney"[All Fields] AND "transplantation"[All Fields]) OR "Kidney Transplantation"[All Fields] OR ("kidney"[All Fields] AND "grafting"[All Fields]) OR "kidney grafting"[All Fields]) OR ("Kidney Transplantation"[MeSH Terms] OR ("kidney"[All Fields] AND "transplantation"[All Fields]) OR "Kidney Transplantation"[All Fields] OR ("transplantation"[All Fields] AND "kidney"[All Fields]) OR "transplantation kidney"[All Fields]) OR ("Kidney Transplantation"[MeSH Terms] OR ("kidney"[All Fields] AND "transplantation"[All Fields]) OR "Kidney Transplantation"[All Fields] OR ("kidney"[All Fields] AND "transplantations"[All Fields]) OR "kidney transplantations"[All Fields]) OR ("Kidney Transplantation"[MeSH Terms] OR ("kidney"[All Fields] AND "transplantation"[All Fields]) OR "Kidney Transplantation"[All Fields] OR ("transplantations"[All Fields] AND "kidney"[All Fields]) OR "transplantations kidney"[All Fields]))) |
| **Cochrane Library and Embase (via OvidSP)** | ("kidney transplantation/" OR "Renal Transplantation.mp." OR "Renal Transplantations.mp." OR "Transplantations, Renal.mp." OR "Transplantation, Renal.mp." OR "Grafting, Kidney.mp." OR "Kidney Grafting.mp." OR "Transplantation, Kidney.mp." OR "Kidney Transplantations.mp." OR "Transplantations, Kidney.mp.") AND ("immunoglobulin A nephropathy/" OR "Glomerulonephritides, IGA.mp." OR "Berger's Disease.mp." OR "Bergers Disease.mp." OR "IGA Glomerulonephritis.mp." OR "Nephropathy, IGA.mp." OR "Iga Nephropathy 1.mp." OR "Nephropathy 1, Iga.mp." OR "Immunoglobulin A Nephropathy.mp." OR "Nephropathy, Immunoglobulin A.mp." OR "Nephritis, IGA Type.mp." OR "IGA Type Nephritis.mp." OR "Berger Disease.mp." OR "IGA Nephropathy.mp.") |
| **Web of science** | #1: TS= ("kidney transplantation" OR "Renal Transplantation" OR "Renal Transplantations" OR "Transplantations, Renal" OR "Transplantation, Renal" OR "Grafting, Kidney" OR "Kidney Grafting" OR "Transplantation, Kidney" OR "Kidney Transplantations" OR "Transplantations, Kidney")  #2: TS= ("Glomerulonephritis, IGA" OR "Glomerulonephritides, IGA" OR "Berger's Disease" OR "Bergers Disease" OR "IGA Glomerulonephritis" OR "Nephropathy, IGA" OR "Iga Nephropathy 1" OR "Nephropathy 1, Iga" OR "Immunoglobulin A Nephropathy" OR "Nephropathy, Immunoglobulin A" OR "Nephritis, IGA Type" OR "IGA Type Nephritis" OR "Berger Disease" OR "IGA Nephropathy")  #1 AND #2 |
| **Scopus** | ( ( TITLE-ABS-KEY ( glomerulonephritis, AND iga ) ) OR ( TITLE-ABS-KEY ( glomerulonephritides, AND iga ) ) OR ( TITLE-ABS-KEY ( berger's AND disease ) ) OR ( TITLE-ABS-KEY ( bergers AND disease ) ) OR ( TITLE-ABS-KEY ( iga AND glomerulonephritis ) ) OR ( TITLE-ABS-KEY ( nephropathy, AND iga ) ) OR ( TITLE-ABS-KEY ( iga AND nephropathy 1 ) ) OR ( TITLE-ABS-KEY ( nephropathy AND 1, AND iga ) ) OR ( TITLE-ABS-KEY ( immunoglobulin AND a AND nephropathy ) ) OR ( TITLE-ABS-KEY ( nephropathy, AND immunoglobulin AND a ) ) OR ( TITLE-ABS-KEY ( nephritis, AND iga AND type ) ) OR ( TITLE-ABS-KEY ( iga AND type AND nephritis ) ) OR ( TITLE-ABS-KEY ( berger AND disease ) ) OR ( TITLE-ABS-KEY ( iga AND nephropathy ) ) ) AND ( ( TITLE-ABS-KEY ( kidney AND transplantation ) ) OR ( TITLE-ABS-KEY ( renal AND transplantation ) ) OR ( TITLE-ABS-KEY ( renal AND transplantations ) ) OR ( TITLE-ABS-KEY ( transplantations, AND renal ) ) OR ( TITLE-ABS-KEY ( transplantation, AND renal ) ) OR ( TITLE-ABS-KEY ( grafting, AND kidney ) ) OR ( TITLE-ABS-KEY ( kidney AND grafting ) ) OR ( TITLE-ABS-KEY ( transplantation, AND kidney ) ) OR ( TITLE-ABS-KEY ( kidney AND transplantations ) ) OR ( TITLE-ABS-KEY ( transplantations, AND kidney ) ) ) |
| **CNKI, CBM, WanFang, and VIP** | ((主题=Ig A肾病) OR (主题=免疫球蛋白A肾病)) AND (主题=肾移植) |

**Supplementary Table 2.** The reasons for the exclusion of full-text articles

| **Study** | **Reasons** |
| --- | --- |
| Zhang (2019)^1^ | To investigate the prognosis of patients undergoing KT due to IgAN. No recurrence was involved. |
| Yu (2012)^2^ | To study post-transplant glomerular disease, no IgAN subgroup data were available. |
| Yabu (2011)^3^ | To study post-transplant glomerular disease, no IgAN subgroup data were available. |
| Van Stralen (2013)^4^ | To study glomerular disease recurrence after KT, no IgAN subgroup data were available. |
| Toledo (2011)^5^ | To study glomerular disease recurrence after KT, no IgAN subgroup data were available. |
| Tang (2008)^6^ | Whether the IgAN was recurrent or de novo could not be determined. |
| Suzuki (2000)^7^ | No detailed data. |
| Sumethkul (2001)^8^ | The control was native IgAN. |
| Spinner (2015)^9^ | To investigate the treatment of recurrent glomerulonephritis after KT. |
| Soler (2005)^10^ | No detailed data. |
| Sofue (2015)^11^ | Whether the IgAN was recurrent or de novo could not be determined. |
| Singh (2019)^12^ | To study glomerular disease recurrence after KT, no IgAN subgroup data were available. |
| Schwarz (1991)^13^ | Whether the IgAN was recurrent or de novo could not be determined. |
| Sato (2014)^14^ | With overlapping cohorts. |
| Sakurai (1999)^15^ | To investigate the prognosis of patients undergoing KT due to IgAN. No recurrence was involved. |
| Pruthi (2016)^16^ | To study the prognosis of different primary glomerular diseases after transplantation. |
| Pippias (2016)^17^ | To study the prognosis of different primary glomerular diseases after transplantation. |
| Pham (2012)^18^ | To study post-transplant glomerular disease, no IgAN subgroup data were available. |
| Park (2000)^19^ | To investigate the prognosis of patients undergoing KT due to IgAN. No recurrence was involved. |
| Park (2019)^20^ | To investigate the treatment of recurrent IgAN after KT. |
| Park (2019)^21^ | Whether the IgAN was recurrent or de novo could not be determined. |
| Ostrowska (2007)^22^ | To study post-transplant glomerular disease, no IgAN subgroup data were available. |
| Ohmacht (1997)^23^ | Recurrence was defined as histological recurrence with dysfunction. |
| O'Meara (1989)^24^ | To study post-transplant recurrent glomerular disease, no IgAN subgroup data were available. |
| Nihei (2017)^25^ | To investigate the treatment of recurrent IgAN after KT. |
| Neumayer (1993)^26^ | To study post-transplant glomerular disease, no IgAN subgroup data were available. |
| Neil (2006)^27^ | Excluded study type: review. |
| Mousson (2007)^28^ | No detailed data. |
| Moron (2014)^29^ | To study post-transplant recurrent glomerular disease, no IgAN subgroup data were available. |
| Mirioglu (2017)^30^ | To study post-transplant glomerular disease, no IgAN subgroup data were available. |
| Messina (2016)^31^ | To investigate the treatment of IgAN after KT. |
| Matsukuma (2018)^32^ | To investigate the treatment of IgAN after KT. |
| Mathew (1975)^33^ | Excluded study type: case series. |
| Manuel Cazorla-López (2020)^34^ | Whether the IgAN was recurrent or de novo could not be determined. |
| Lim (2018)^35^ | To study post-transplant glomerular disease, no IgAN subgroup data were available. |
| Lim (2013)^36^ | Whether the IgAN was recurrent or de novo could not be determined. |
| Lemes-Canuto1 (2015)^37^ | To investigate the [characteristic](javascript:;)s of post-transplant IgAN. |
| Leeaphorn (2018)^38^ | To investigate the risk of graft loss attributable to recurrence of IgAN after KT. |
| Kiattisunthorn (2008)^39^ | Whether the IgAN was recurrent or de novo could not be determined. |
| Kennoki (2009)^40^ | To investigate the treatment of recurrent IgAN after KT. |
| Kawabe (2020)^41^ | With overlapping cohorts. |
| Kaneko (2013)^42^ | To investigate the treatment of IgAN after KT. |
| Kadiyala (2015)^43^ | To investigate the prognosis of patients undergoing KT due to IgAN. No recurrence was involved. |
| Joshi (2007)^44^ | To study post-transplant recurrent glomerular disease, no IgAN subgroup data were available. |
| Jeong (2016)^45^ | Whether the IgAN was recurrent or de novo could not be determined. |
| Jeong (2004)^46^ | Whether the IgAN was recurrent or de novo could not be determined. |
| Jeong (2003)^47^ | Whether the IgAN was recurrent or de novo could not be determined. |
| Jeong (2003)^48^ | To investigate the [characteristic](javascript:;)s of recurrent IgAN after KT. |
| Hotta (2013)^49^ | To investigate the treatment of recurrent IgAN after KT. |
| Hariharan (1999)^50^ | To study post-transplant glomerular disease, no IgAN subgroup data were available. |
| Hariharan (1998)^51^ | To study post-transplant glomerular disease, no IgAN subgroup data were available. |
| Hariharan (2000)^52^ | Excluded study type: review. |
| Guo (2014)^53^ | To investigate the prognosis of patients undergoing KT due to IgAN. No recurrence was involved. |
| Freese (1997)^54^ | To investigate the prognosis of patients undergoing KT due to IgAN. No recurrence was involved. |
| Floege (2014)^55^ | To study post-transplant recurrent glomerular disease, no IgAN subgroup data were available. |
| Figueiredo Júnior (2000)^56^ | To study post-transplant recurrent glomerular disease, no IgAN subgroup data were available. |
| Eder (2021)^57^ | Whether the IgAN was recurrent or de novo could not be determined. |
| Cabral (2018)^58^ | Whether the IgAN was recurrent or de novo could not be determined. |
| Coppo (1995)^59^ | With overlapping cohorts. |
| Clayton (2011)^60^ | To investigate the risk of graft loss attributable to recurrence of IgAN after KT. |
| Chancharoenthana (2021)^61^ | To investigate the treatment of recurrent IgAN after KT. |
| Cazorla-López (2020)^62^ | Whether the IgAN was recurrent or de novo could not be determined. |
| Cattaneo (2007)^63^ | To investigate the prognosis of patients undergoing KT due to IgAN. No recurrence was involved. |
| Carneiro-Roza (2006)^64^ | To study post-transplant glomerular disease, no IgAN subgroup data were available. |
| Cañas (2015)^65^ | To study post-transplant recurrent glomerular disease, no IgAN subgroup data were available. |
| Berger (1988)^66^ | Excluded study type: case series. |
| Andresdottir (2005)^67^ | To investigate the prognosis of patients undergoing KT due to IgAN. No recurrence was involved. |
| Andresdottir (2009)^68^ | To investigate the prognosis of patients undergoing KT due to IgAN. No recurrence was involved. |
| Huang (2019)^69^ | To study post-transplant glomerular disease, no IgAN subgroup data were available. |
| Chan (2005)^70^ | With overlapping cohorts. |
| Kim (2002)^71^ | With overlapping cohorts. |
| Matsugami (1998)^72^ | Published in Japanese. |
| Andresdottir (2001)^73^ | Full text was not accessible. |
| Frohnert (1997)^74^ | Full text was not accessible. |
| Bantis (2007)^75^ | Published in German. |

**Abbreviation:** KT, kidney transplantation; IgAN, immunoglobulin A nephropathy.

**Reference**

1. Zhang J, Chen G-d, Qiu J, et al. Graft failure of IgA nephropathy in renal allografts following living donor transplantation: predictive factor analysis of 102 biopsies. Bmc Nephrology. 2019;20(1).

2. Yu T-M, Wen M-C, Wu M-J, et al. Impact of Posttransplantation Glomerulonephritis on Long-term Outcome of Kidney Transplants: Single-Center 20-Year Experience. World Journal of Surgery. 2012;36(12):2923-2930.

3. Yabu JM, Higgins JP, Chen G, Sequeira F, Busque S, Tyan DB. C1q-Fixing Human Leukocyte Antigen Antibodies Are Specific for Predicting Transplant Glomerulopathy and Late Graft Failure After Kidney Transplantation. Transplantation. 2011;91(3):342-347.

4. Van Stralen KJ, Verrina E, Belingheri M, et al. Impact of graft loss among kidney diseases with a high risk of post-transplant recurrence in the paediatric population. Nephrology Dialysis Transplantation. 2013;28(4):1031-1038.

5. Toledo K, Perez-Saez MJ, Navarro MD, et al. Impact of Recurrent Glomerulonephritis on Renal Graft Survival. Transplantation Proceedings. 2011;43(6):2182-2186.

6. Tang Z, Ji S-M, Chen D-R, et al. Recurrent or de novo IgA nephropathy with crescent formation after renal transplantation. Renal Failure. 2008;30(6):611-616.

7. Suzuki K, Tanabe K, Tokumoto T, et al. Effect of tacrolimus in renal transplant recipients with immunoglobulin-A nephropathy. Transplant Proc. 2000;32(7):1730-1732.

8. Sumethkul V, Jirasiritham S, Chalermsanyakorn P, Buranachokpaisan W. Chronic rejection: a significant predictor of poor outcome for recurrence IgA nephropathy. Transplant Proc. 2001;33(7-8):3375-3376.

9. Spinner ML, Bowman LJ, Horwedel TA, Delos Santos RB, Klein CL, Brennan DC. Single-dose rituximab for recurrent glomerulonephritis post-renal transplant. American Journal of Nephrology. 2015;41(1):37-47.

10. Soler MJ, Mir M, Rodriguez E, et al. Recurrence of IgA nephropathy and Henoch-Schönlein purpura after kidney transplantation: risk factors and graft survival. Transplant Proc. 2005;37(9):3705-3709.

11. Sofue T, Inui M, Hara T, et al. Association between post-transplantation immunoglobulin A deposition and reduced allograft function. Transplant Proc. 2015;47(2):332-336.

12. Singh T, Astor BC, Zhong W, Mandelbrot DA, Maursetter L, Panzer SE. The association of acute rejection vs recurrent glomerular disease with graft outcomes after kidney transplantation. Clin Transplant. 2019;33(12):e13738.

13. Schwarz A, Krause PH, Offermann G, Keller F. Recurrent and de novo renal disease after kidney transplantation with or without cyclosporine A. Am J Kidney Dis. 1991;17(5):524-531.

14. Sato Y, Ishida H, Shimizu T, Tanabe K. Evaluation of tonsillectomy before kidney transplantation in patients with IgA nephropathy. Transpl Immunol. 2014;30(1):12-17.

15. Sakurai T, Okamoto N, Fukazawa S, Sirosita K, Ueda T, Hirano T. Is IgA nephropathy in renal transplants a risk leading to graft failure? Transplant Proc. 1999;31(6):2651-2654.

16. Pruthi R, McClure M, Casula A, et al. Long-term graft outcomes and patient survival are lower posttransplant in patients with a primary renal diagnosis of glomerulonephritis. Kidney Int. 2016;89(4):918-926.

17. Pippias M, Stel VS, Aresté-Fosalba N, et al. Long-term Kidney Transplant Outcomes in Primary Glomerulonephritis: Analysis From the ERA-EDTA Registry. Transplantation. 2016;100(9):1955-1962.

18. Pham PTT, Pham PCT. The impact of mycophenolate mofetil versus azathioprine as adjunctive therapy to cyclosporine on the rates of renal allograft loss due to glomerular disease recurrence. Nephrology Dialysis Transplantation. 2012;27(7):2965-2971.

19. Park SB, Kim HC, Kim HT, Cho WH, Park CH, Park KK. Long-term outcome of IgA nephropathy in living related kidney transplantation. Transplant Proc. 2000;32(7):1828-1830.

20. Park S, Baek CH, Go H, et al. Possible beneficial association between renin-angiotensin-aldosterone-system blockade usage and graft prognosis in allograft IgA nephropathy: a retrospective cohort study. Bmc Nephrology. 2019;20(1).

21. Park S, Baek CH, Cho H, et al. Glomerular crescents are associated with worse graft outcome in allograft IgA nephropathy. American Journal of Transplantation. 2019;19(1):145-155.

22. Ostrowska J, Pazik J, Lewandowski Z, Mrdz A, Perkowska-Ptasinska A, Durlik M. Posttransplantation glomerulonephritis: Risk factors associated with kidney allograft loss. Transplantation Proceedings. 2007;39(9):2751-2753.

23. Ohmacht C, Kliem V, Burg M, et al. Recurrent immunoglobulin A nephropathy after renal transplantation: a significant contributor to graft loss. Transplantation. 1997;64(10):1493-1496.

24. O'Meara Y, Green A, Carmody M, et al. Recurrent glomerulonephritis in renal transplants: fourteen years' experience. Nephrol Dial Transplant. 1989;4(8):730-734.

25. Nihei H, Sakai K, Shishido S, Sibuya K, Edamatsu H, Aikawa A. Efficacy of tonsillectomy for the treatment of immunoglobulin A nephropathy recurrence after kidney transplantation. Ren Replace Ther. 2017;3(1).

26. Neumayer HH, Kienbaum M, Graf S, Schreiber M, Mann JF, Luft FC. Prevalence and long-term outcome of glomerulonephritis in renal allografts. Am J Kidney Dis. 1993;22(2):320-325.

27. Neil D. Recurrent and de novo disease in kidney, heart, lung, pancreas and intestinal transplants. Current Opinion in Organ Transplantation. 2006;11(3):289-295.

28. Mousson C, Charon-Barra C, de la Vega MF, et al. Recurrence of IgA Nephropathy With Crescents in Kidney Transplants. Transplantation Proceedings. 2007;39(8):2595-2596.

29. Moroni G, Longhi S, Quaglini S, et al. The impact of recurrence of primary glomerulonephritis on renal allograft outcome. Clinical Transplantation. 2014;28(3):368-376.

30. Mirioglu S, Caliskan Y, Goksoy Y, et al. Recurrent and de novo glomerulonephritis following renal transplantation: higher rates of rejection and lower graft survival. Int Urol Nephrol. 2017;49(12):2265-2272.

31. Messina M, di Vico MC, Ariaudo C, et al. Treatment protocol with pulse and oral steroids for IgA Nephropathy after kidney transplantation. J Nephrol. 2016;29(4):575-583.

32. Matsukuma Y, Masutani K, Tsuchimoto A, et al. Effect of steroid pulse therapy on post-transplant immunoglobulin A nephropathy. Nephrology (Carlton). 2018;23 Suppl 2:10-16.

33. Mathew TH, Mathews DC, Hobbs JB, Kincaid-Smith P. Glomerular lesions after renal transplantation. Am J Med. 1975;59(2):177-190.

34. Manuel Cazorla-Lopez J, Wu J, Villanego-Fernandez F, et al. IgA Nephropathy After Renal Transplant: Recurrences and De Novo Cases. Transplantation Proceedings. 2020;52(2):515-518.

35. Lim WH, Wong G, McDonald SP, et al. Long-term outcomes of kidney transplant recipients with end-stage kidney disease attributed to presumed/advanced glomerulonephritis or unknown cause. Sci Rep. 2018;8(1):9021.

36. Lim BJ, Joo DJ, Kim MS, et al. Usefulness of Oxford classification in assessing immunoglobulin A nephropathy after transplantation. Transplantation. 2013;95(12):1491-1497.

37. Lemes-Canuto AP, de Sandes-Freitas TV, Medina-Pestana JO, Mastroianni-Kirsztajn G. IgA NEPHROPATHY IN PATIENTS RECEIVING A RENAL TRANSPLANT. J Ren Care. 2015;41(4):222-230.

38. Leeaphorn N, Garg N, Khankin EV, Cardarelli F, Pavlakis M. Recurrence of IgA nephropathy after kidney transplantation in steroid continuation versus early steroid-withdrawal regimens: a retrospective analysis of the UNOS/OPTN database. Transpl Int. 2018;31(2):175-186.

39. Kiattisunthorn K, Premasathian N, Wongwiwatana A, Parichatikanond P, Cheunsuchon B, Vasuvattakul S. Evaluating the clinical course and prognostic factors of posttransplantation immunoglobulin A nephropathy. Transplant Proc. 2008;40(7):2349-2354.

40. Kennoki T, Ishida H, Yamaguchi Y, Tanabe K. Proteinuria-reducing effects of tonsillectomy alone in IgA nephropathy recurring after kidney transplantation. Transplantation. 2009;88(7):935-941.

41. Kawabe M, Yamamoto I, Yamakawa T, et al. Association Between Galactose-Deficient IgA1 Derived From the Tonsils and Recurrence of IgA Nephropathy in Patients Who Underwent Kidney Transplantation. Front Immunol. 2020;11:2068.

42. Kaneko T, Shimizu A, Tsuruoka S, Iino Y, Katayama Y. Efficacy of Steroid Pulse Therapy in Combination with Mizoribine Following Tonsillectomy for Immunoglobulin A Nephropathy in Renally Impaired Patients. Journal of Nippon Medical School. 2013;80(4):279-286.

43. Kadiyala A, Mathew AT, Sachdeva M, et al. Outcomes following Kidney transplantation in IgA nephropathy: a UNOS/OPTN analysis. Clin Transplant. 2015;29(10):911-919.

44. Joshi K, Nada R, Minz M, Sakhuja V. Recurrent glomerulopathy in the renal allograft. Transplant Proc. 2007;39(3):734-736.

45. Jeong HS, Lee J, Lim BJ, et al. Concurrent Post-Transplantation Diabetes Mellitus in Renal Allograft Recipients With Immunoglobulin A Nephropathy. Transplant Proc. 2016;48(3):887-889.

46. Jeong HJ, Kim YS, Kwon KW, et al. Segmental glomerulosclerosis in IgA nephropathy after renal transplantation: Relationship with proteinuria and therapeutic response to enalapril. Clinical Transplantation. 2003;17(2):108-113.

47. Jeong HJ, Kim YS, Kwon KH, et al. Glomerular crescents are responsible for chronic graft dysfunction in post-transplant IgA nephropathy. Pathol Int. 2004;54(11):837-842.

48. Jeong HJ, Hong SW, Kim YS, et al. Histologic factors associated with nephrotic-range proteinuria in recurrent IGA nephropathy. Transplant Proc. 2003;35(1):291.

49. Hotta K, Fukasawa Y, Akimoto M, et al. Tonsillectomy ameliorates histological damage of recurrent immunoglobulin A nephropathy after kidney transplantation. Nephrology (Carlton). 2013;18(12):808-812.

50. Hariharan S, Adams MB, Brennan DC, et al. Recurrent and de novo glomerular disease after renal transplantation: A report from renal allograft disease registry. Transplantation Proceedings. 1999;31(1-2):223-224.

51. Hariharan S, Peddi VR, Savin VJ, et al. Recurrent and de novo renal diseases after renal transplantation: a report from the renal allograft disease registry. Am J Kidney Dis. 1998;31(6):928-931.

52. Hariharan S. Recurrent and de nova diseases after renal transplantation. Seminars in Dialysis. 2000;13(3):195-199.

53. Guo JQ, Song BL, Wu ZX, et al. Prognostic factors for renal allograft survival in patients with immunoglobulin A nephropathy: a case control study. Mol Med Rep. 2014;9(4):1179-1184.

54. Freese P, Nordén G, Olausson M, Nyberg G. Rejection rates in kidney transplant patients with and without IgA nephropathy. Nephrol Dial Transplant. 1997;12(11):2385-2388.

55. Floege J, Regele H, Gesualdo L, Working E-EI. The ERA-EDTA database on recurrent glomerulonephritis following renal transplantation. Nephrology Dialysis Transplantation. 2014;29(1):15-21.

56. Figueiredo Junior HE, Nascimento E, Lasmar MF, Siqueira RG, Sousa RN, Fabreti-Oliveira RA. Effect of Glomerulopathy Recurrence in the Outcome and Graft Survival of Kidney Transplanted Patients. Transplantation Proceedings. 2020;52(5):1272-1278.

57. Eder M, Kozakowski N, Omic H, et al. Glomerular C4d in Post-Transplant IgA Nephropathy is associated with decreased allograft survival. J Nephrol. 2021;34(3):839-849.

58. Cordeiro Cabral DB, de Sandes-Freitas TV, Medina-Pestana JO, Mastroianni-Kirsztajn G. Clinical Features, Treatment and Prognostic Factors of Post-Transplant Immunoglobulin A Nephropathy. Ann Transplant. 2018;23:166-175.

59. Coppo R, Amore A, Cirina P, et al. IgA serology in recurrent and non-recurrent IgA nephropathy after renal transplantation. Nephrol Dial Transplant. 1995;10(12):2310-2315.

60. Clayton P, McDonald S, Chadban S. Steroids and recurrent IgA nephropathy after kidney transplantation. Am J Transplant. 2011;11(8):1645-1649.

61. Chancharoenthana W, Leelahavanichkul A, Ariyanon W, Vadcharavivad S, Phumratanaprapin W. Comparative Long-Term Renal Allograft Outcomes of Recurrent Immunoglobulin A with Severe Activity in Kidney Transplant Recipients with and without Rituximab: An Observational Cohort Study. J Clin Med. 2021;10(17).

62. Cazorla-López JM, Wu J, Villanego-Fernández F, et al. IgA Nephropathy After Renal Transplant: Recurrences and De Novo Cases. Transplant Proc. 2020;52(2):515-518.

63. Cattaneo D. Does immunoglobulin A nephropathy affect long-term graft outcome after kidney transplantation? J Postgrad Med. 2007;53(2):84.

64. Carneiro-Roza F, Medina-Pestana JO, Moscoso-Solorzano G, Franco M, Ozaki K, Mastroianni-Kirsztajn G. Initial response to immunosuppressive and renoprotective treatment in posttransplant glomerulonephritis. Transplant Proc. 2006;38(10):3491-3497.

65. Cañas L, López D, Pérez JF, et al. Recurrent Glomerulonephritis in Renal Transplantation: Experience in Our Renal Transplantation Center. Transplant Proc. 2015;47(8):2354-2356.

66. Berger J. Recurrence of IgA nephropathy in renal allografts. Am J Kidney Dis. 1988;12(5):371-372.

67. Andresdottir MB, Haasnoot GW, Doxiadis IIN, Persijn GG, Claas FHJ. Exclusive characteristics of graft survival and risk factors in recipients with immunoglobulin A nephropathy: A retrospective analysis of registry data. Transplantation. 2005;80(8):1012-1018.

68. Andresdottir MB, Haasnoot GW, Persijn GG, Claas FH. HLA-B8, DR3: a new risk factor for graft failure after renal transplantation in patients with underlying immunoglobulin A nephropathy. Clin Transplant. 2009;23(5):660-665.

69. Huang Y, Fan L, Deng S, Shen M, Zhang W, Chen S: Analysis of survival and influencing factors of patients with recurrent and de novo nephritis of renal allograft. Organ Transplantation. 2019;10(1):67-73.

70. Chan KW, Chan GSW, Tang S. Glomerular pathology of allograft kidneys in Hong Kong. Transplantation Proceedings. 2005;37(10):4293-4296.

71. Kim YS, Jeong HJ, Kwon KW, Lee HY, Han DS, Park K. Live donor renal transplantation in patients with end-stage renal failure due to IgA nephropathy: clinicopathological assessment. Nephrology. 2002;7:S74-S77.

72. Matsugami K, Naito T, Nitta K, et al. [A clinicopathological study of recurrent IgA nephropathy following renal transplantation]. Nihon Jinzo Gakkai Shi. 1998;40(5):322-328.

73. Andresdottir MB, Hoitsma AJ, Assmann KJ, Wetzels JF. Favorable outcome of renal transplantation in patients with IgA nephropathy. Clin Nephrol. 2001;56(4):279-288.

74. Frohnert PP, Donadio JV, Jr., Velosa JA, Holley KE, Sterioff S. The fate of renal transplants in patients with IgA nephropathy. Clin Transplant. 1997;11(2):127-133.

75. Bantis C, Heering PJ, Blume C, et al. Recurrence of IgA nephropathy after renal transplantation. [German]. Nieren- und Hochdruckkrankheiten. 2007;36(3):81-86.
